# Supplementary material for: A network dynamical simulation model for the study of antibiotic resistance in microbial communities
Source: Gut Microbes. 2026 Sep 19;18(1):2734703. doi: 10.1080/19490976.2026.2734703 (PMC13596919; doi:10.1080/19490976.2026.2734703)
Supplement: Supplementary file 1 [file KGMI_A_2734703_SM9053.zip › supplementary_file_1/Figures/ba_delta_gc_frac_n300.pdf]

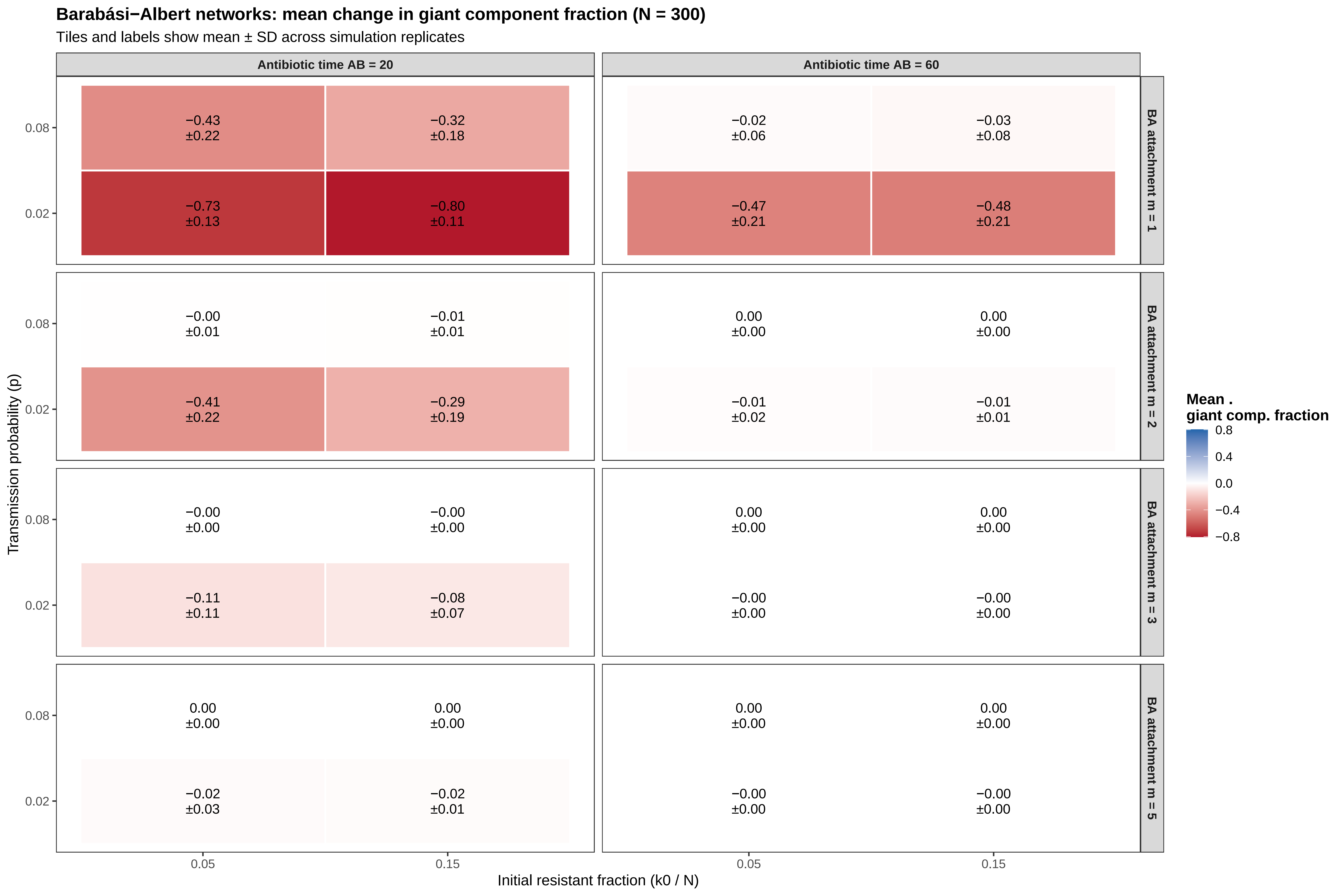

Barabási−Albert networks: mean change in giant component fraction (N = 300)
Tiles and labels show mean ± SD across simulation replicates
Antibiotic time AB = 20
Antibiotic time AB = 60
BA attachment m = 1
BA attachment m = 2
BA attachment m = 3
BA attachment m = 5
−0.43 ±0.22
−0.32 ±0.18
−0.73 ±0.13
−0.80 ±0.11
−0.02 ±0.06
−0.03 ±0.08
−0.47 ±0.21
−0.48 ±0.21
−0.00 ±0.01
−0.01 ±0.01
−0.41 ±0.22
−0.29 ±0.19
0.00 ±0.00
0.00 ±0.00
−0.01 ±0.02
−0.01 ±0.01
−0.00 ±0.00
−0.00 ±0.00
−0.11 ±0.11
−0.08 ±0.07
0.00 ±0.00
0.00 ±0.00
−0.00 ±0.00
−0.00 ±0.00
0.00 ±0.00
0.00 ±0.00
−0.02 ±0.03
−0.02 ±0.01
0.00 ±0.00
0.00 ±0.00
−0.00 ±0.00
−0.00 ±0.00
Transmission probability (p)
0.08
0.02
Initial resistant fraction (k0 / N)
0.05
0.15
Mean .
giant comp. fraction
0.8
0.4
0.0
−0.4
−0.8
